# Nitrogen in the Environment: Sources, Problems, and Management

**DOI:** 10.1100/tsw.2001.269

**Published:** 2001-10-30

**Authors:** R.F. Follett, J.L. Hatfield

**Affiliations:** USDA-ARS, Soil-Plant-Nutrient Unit, Fort Collins, Co 80522, USA

**Keywords:** soil, plants, N-cycle, greenhouse gases, human N-requirement, hydrology, marine systems, fertilizer, livestock, manure, drinking water quality, ecosystems, streams, rivers, policy

## Abstract

Nitrogen (N) is applied worldwide to produce food. It is in the atmosphere, soil, and water and is essential to all life. N for agriculture includes fertilizer, biologically fixed, manure, recycled crop residue, and soil-mineralized N. Presently, fertilizer N is a major source of N, and animal manure N is inefficiently used. Potential environmental impacts of N excreted by humans are increasing rapidly with increasing world populations. Where needed, N must be efficiently used because N can be transported immense distances and transformed into soluble and/or gaseous forms that pollute water resources and cause greenhouse effects. Unfortunately, increased amounts of gaseous N enter the environment as N_2_O to cause greenhouse warming and as NH_3_ to shift ecological balances of natural ecosystems. Large amounts of N are displaced with eroding sediments in surface waters. Soluble N in runoff or leachate water enters streams, rivers, and groundwater. High-nitrate drinking water can cause methemoglobinemia, while nitrosamines are associated with various human cancers. We describe the benefits, but also how N in the wrong form or place results in harmful effects on humans and animals, as well as to ecological and environmental systems.

### INTRODUCTION

Nitrogen (N) is among the most important elements required in agricultural systems to produce plant and animal products for the increasing world population. The world's population is projected to increase from current levels of about 6.1 to nearly 8 billion people during the next 25 years[1], and the requirement for N must increase to meet this population growth. Countries such as China are rapidly increasing the use of N fertilizer as a result of their large population and an increased demand for dietary protein, including animal protein. Such a trend should be expected and planned for in many other parts of the world with growing populations and increased demands for improved living standards. Increasing human populations are the driving force for increased production of dietary protein, N inputs into crop and livestock production systems, and livestock and human-excretory N contributions to the environment.

Much of this manuscript is based upon information developed for a book[2] by the authors of this article, and whose overall objective is to address the potential needs and consequences of the increase in N inputs and their real and potential impacts upon the environment as human populations and needs increase in the future. It is necessary that policy makers, industry, the public, and producers be aware of the emerging issues that will result. These issues will require a strategy that encompasses both policy and science during the next decades in order to develop viable solutions. It will be important that a multidisciplinary approach be used to holistically address the topic of N in the environment, the sources of N, and the issues associated with its management. Policymakers, joined by experts from many disciplines that can address both the agricultural production as well as the environmental issues, and other interested stakeholders must become increasingly engaged in a sustained discussion to address the multifaceted issues that are involved.

### NITROGEN, AGRICULTURE, AND HUMAN DIETS

#### Agricultural Nitrogen Use

N is ubiquitous in the environment. It is one of the most important nutrients and is required for the survival of all living things. It is also central to the production of all crop plants. N accounts for 78% of the atmosphere as elemental $N_2$ gas. Elemental $N_2$ gas is inert, does not impact environmental quality, and is not directly available for plant uptake and metabolism. However, during the past 4 decades unprecedented amounts of reactive N have been added into terrestrial systems[3]. These inputs of reactive N, termed here as $NH_x$ (ammonia [$NH_3$] and ammonium [$NH_4^+$]), $NO_x$ (nitric oxide [NO] + nitrogen dioxide [$NO_2$]) and nitrate ($NO_3^-$) and nitrite ($NO_2^-$), are occurring through increased biological fixation of atmospheric $N_2$, combustion of fossil fuels, and production of synthetic fertilizer N[4,5]. Until the 1970s, most of the industrial N fertilizer was used in developed countries. Since then, use in developed countries has stabilized or declined, while it has increased in developing countries. Estimates[6] are that globally human activities cause the fixation of about 150 Tg N year$^{-1}$. Of this total, about 57% is as N fertilizer, 29% is from leguminous crops, and 14% is from fossil fuel. There is an additional release of reactive N from stable soil organic matter as a result of soil disturbance for crop production that is not readily quantifiable.

Available soil N supplies are often inadequate for optimum crop production. Crop N needs can usually be met because commercial fertilizer, manures, and other sources of N are generally easily and economically applied. An important consideration to help minimize losses of reactive N into the environment is to keep applied and residual sources of N within the soil-crop system by curtailing transport processes (leaching, runoff, erosion, and gaseous losses) that carry N into the surrounding environment. The objective is to lower the rate and duration of the loss processes themselves. Evaluation of the transport and fate of N through agricultural and other systems must be done within the concept of the N cycle. Can the N cycle be managed to avoid some of the problems that N generates? Given the world's need for food, the great ability of annual grains to produce the needed food (including animal feed), and the relative cheapness of N fertilizer due to undervalued energy, changes to the unprecedented amounts of reactive N that are being added into terrestrial systems and deceases in the use of excess inputs of N on the larger scale do not seem to be forthcoming. In order to develop strategies that improve the efficiency of N fertilizer use, the transport and flow of N through agricultural and into natural ecosystems needs to be reduced and the flow of N needs to be budgeted as it is followed through the pathways of the N cycle at farms, watersheds, river basins, and even larger scales.

#### Nitrogen in Human Diets

N greatly influences the amounts and types of dietary proteins and amino acids available for human nutrition and health from major crops. Humans are generally not able to utilize more simple forms of N that they are exposed to for normal physiological functions such as energy and synthesis of proteins. However, humans absorb such forms of N as $NO_3^-$ and $NO_2^-$. Nitrate can be obtained from plant sources as well as from contaminated drinking water and is easily absorbed by the intestine. The main health risk associated with $NO_3^-$ consumption is methemoglobinemia, due to the conversion of $NO_3^-$ to $NO_2^-$, which in turn can interact with hemoglobin, thus leading to formation of methemoglobin, which then leads to oxygen deprivation of the cells. Secondary and related deleterious effects of exposure to $NO_3^-$ include increased respiratory infections, inhibition of iodine uptake by the thyroid, and possible reproductive problems. There is also growing acceptance for the beneficial effects of the related compound nitric oxide (NO). NO is a free radical gas that acts as a messenger molecule for regulation of several systems including blood vessel dilation and hormonal and neurotransmission functions. Another group of N-containing substances are N-nitroso compounds (NOC), which include nitrosamines and nitrosamides. These compounds are important to consider in the human diet since they are capable of participating in DNA alkylation and appear to be among the most potent and broad-acting carcinogens known.

### TRANSPORT OF NITROGEN BY WATER

Water in the form of precipitation, flowing across the soil surface as runoff, and percolating through soil layers to groundwater is a significant carrier of organic and inorganic N. The relative importance of these transport mechanisms is a complex function of N sources and transformations, hydrologic processes, climate patterns, and land use.

#### Farm Scale

Rate of N application is the nutrient management practice that most influences N losses at the field scale. Time of N application and nitrification inhibitors play a significant role in minimizing $NO_3^-$ loss, especially between crops under wet and warm conditions. Tillage systems generally have little effect on $NO_3^-$ losses except for fallow periods and when disturbance promotes mineralization and leaching. Tile drainage is a common water management practice in highly productive agricultural areas with poorly drained soils that have seasonally perched water tables or shallow groundwater. Factors influencing the $NO_3^-$ content in subsurface waters draining from agricultural production landscapes can be divided into two categories: uncontrollable and controllable. Precipitation, including variations in annual amount, temporal distribution within a year, or extreme daily events, is an uncontrollable factor with a large effect on $NO_3^-$ loss. Mineralization of soil-organic N is an uncontrollable process, but one that can be manipulated; it produces $NO_3^-$, which can be lost in drainage if crop uptake is not synchronized with release of the $NO_3^-$. Controllable factors are the management practices that crop producers use to improve yield and profitability of their enterprise. Cropping system is the controllable factor that has the greatest effect on $NO_3^-$ losses. Limited field studies have shown decreased tile spacing to increase losses, although modeling efforts suggest a minimal effect of spacing and tile depth. Water table management, through controlled drainage systems, significantly affects $NO_3^-$ losses. This management practice increases crop productivity, reduces risk, and improves economic returns to crop producers. Such practices are also a source of N to surface waters. The occurrence of N in groundwater beneath agricultural systems requires an examination of the factors influencing aquifer vulnerability to N contamination and the characterization of the geographic distribution of groundwater contamination by N.

### Watershed Scale

Movement of N in the terrestrial environment is intimately related to the movement of water. Water in the form of precipitation, its movement as overland and interflow as runoff, and percolation through soil layers to ground water are all significant. While some elements of the N cycle can be studied in the laboratory under controlled experimental conditions, many can only be studied in a meaningful way in the natural and culturally affected environments of watersheds. Factors that influence aquifer vulnerability must be considered in the context of exposure to N sources from general agricultural systems and hydrologic conditions that facilitate transfer of those sources to groundwater. From there, the discussion of the transport of N must extend to the role of watersheds. By considering N transport across a range of spatial and temporal scales, it is possible to improve understanding of the factors affecting N fate in watersheds, as well as an understanding of the effects of land use and N sources (point, nonpoint, agricultural, urban, organic, and inorganic), transformation (mineralization, nitrification, denitrification, and immobilization), and transport mechanisms (runoff, percolation to groundwater, and groundwater transport). Knowledge of the variability in N transport in relation to these factors is critical to developing and implementing effective strategies for mitigating unacceptably high N inputs to receiving waters. The technical issues must include those related to the flow and chemistry of fixed (i.e., biologically reactive) organic and inorganic N forms in watersheds and the use of empirical modeling methods to separate the effects of N supply and loss processes to estimate the fate of N sources in watersheds.

### Riverine Systems

Measures need to be developed to protect and maintain the environmental quality of rivers, lakes, and coastal waters. Elevated riverine-N loading has been associated with increased primary production and nuisance algal growth in coastal zones, semi-enclosed and enclosed areas, and seas. Knowledge of the fate of the riverine-N transport (mostly as $NO_3^-$) is important for allowing accurate estimations of the resulting net release of N to the open sea. The EU countries have identified zones vulnerable to $NO_3^-$ pollution, and most countries have adopted action plans to reduce N pollution. Combating N pollution from both point and diffuse sources is a task requiring thorough knowledge of the mechanisms governing N loss that arises from different uses of land, as well as of the fate of N in ground- and surface waters. A high proportion of total anthropogenic-N loading to the aquatic environment is from agricultural sources. Reduction of agricultural-N loading appears to hold considerable promise even though reducing such inputs may be difficult for both technical and political reasons. Another promising approach to reduce the level of N is to reinstate formerly drained lakes and wetlands that have the potential for $NO_3^-$ removal through denitrification as high $NO_3^-$ waters flow through them. To determine the potential effectiveness of this approach requires quantifying the transformation of $NO_3^-$ under anoxic conditions into nitrous oxide ($N_2O$) and $N_2$ gases in rivers, lakes, wetlands, and estuaries.

### Coastal Marine Environments

The effects of N on coastal marine environments as a result of riverine and atmospheric enrichments, combined with burgeoning coastal zone populations of humans and their associated land-use changes and wastewater releases, make coastal systems a frontline receiving system for much of the enormous global N enrichment that is occurring. Symptoms of excess enrichment of coastal systems have been observed with increasing frequency in more places and at expanding spatial scales since the middle to the end of the 20th century. This may partially be a consequence of greater attention to the problem, but it is also not surprising, given noted increases in N delivery to many coastal systems. Humans and their activities are disproportionately distributed towards coastlines, a factor which has also contributed to increased nutrient inputs, including N, to coastal receiving waters. N inputs to coastal waters come from the land, sea, and air. Because of multiple sources and the complexity of the N cycle, development of complete nutrient budgets and confident estimates of N loading are not routine, a fact which has hindered development of quantitative loading-response relationships that otherwise could aid management. Moreover, there is great diversity in coastal systems (estuaries, small and large embayments, lagoons, open shelfwaters, and semi-enclosed coastal seas), and their vulnerability to increased N loading varies greatly. Nonetheless, the principal ecological problems from increasing inputs of N to coastal waters are known and arise from stimulation of algal growth and productivity. Possible consequential effects include the following: depressed levels of dissolved oxygen (hypoxia and anoxia) that injure or kill sensitive biological species; decline or elimination of submerged aquatic vegetation, which provides critical habitat for some fish and shellfish in shallow areas; promotion of certain algal species that are harmful because they produce toxins; and other undesirable changes that affect coastal food webs and fisheries. Evidence of a causal connection between increased N loading and stimulation of a plankton growth/productivity response is very strong; it comes from many studies of individual systems over time and from comparisons of different systems with different N input rates, and it is confirmed by controlled enrichment experiments.

## ATMOSPHERIC TRANSPORT OF NITROGEN

### Livestock Systems

On a global scale, livestock farming systems contribute about 70% and about 30% to the total anthropogenic emissions into the atmosphere of $NH_3$ and $N_2O$, respectively. Basically, livestock farming systems transform carbohydrates and protein from plants into milk, meat, and eggs. Usually not more than 5 to 30% of the plant protein is transformed into animal protein, depending on animal type and management. The remaining 70 to 95% is excreted via urine and dung as organically bound N. Following its deposition on the floor of animal housing systems or in pastures, a large fraction of the organic N is rapidly hydrolyzed into $NH_4^+$. The $NH_4^+$ in urine and dung is conducive to volatilization as $NH_3$. The $NH_4^+$ is also substrate for nitrifying bacteria upon aeration of the manure (dung and urine). Nitrifying bacteria convert $NH_4^+$ into $NO_3^-$, which can be converted subsequently into $N_2$ gas by denitrifying bacteria under anoxic conditions. During the nitrification of $NH_4^+$ and the denitrification of $NO_3^-$, NO, and $N_2O$ may escape into the atmosphere, together with the gaseous $N_2$ from denitrification. The total loss of $NH_3$, NO, $N_2O$, and $N_2$ from animal housing systems and manure storage systems ranges from about 10% of the excreted N in dung and urine from dairy cattle up to more than 30% for pigs and poultry in intensive livestock operations. Another 10 to 50% of total N in the manure may escape as $NH_3$, NO, $N_2O$, and $N_2$ from the soil following application to agricultural land. The uncertainty in these estimates is large because there are many possible sites for gaseous N compounds to escape from the livestock farming system, and there are also many different types of farm animals, livestock farming systems, and manure management systems, while the number of measurements of gaseous N losses is still limited. It is suggested that the emissions of $NH_3$, NO, $N_2O$, and $N_2$ from livestock farming systems will continue to increase because of the increasing quest of animal protein by the growing human population, unless effective mitigation measures are implemented in practice.

### Other Systems

In addition to livestock systems, industrial, automobile, and food production systems are important contributors to local, regional, and global $NH_3$, $NO_x$ (NO + $NO_2$), and $N_2O$ budgets through the emissions mainly of $NO_x$ and $NH_3$ via the atmosphere and the release of $NO_3^-$ into ground- and surface waters. These reactive N species are highly mobile and may cross national boundaries to be deposited as $NO_y$ ($NO_x$ + organic nitrates, inorganic nitrates, and nitrites) and $NH_X$ ($NH_3$ and $NH_4^+$) or $NO_3^-$ far from the source of N. Such emissions into the atmosphere serve to redistribute fixed N to local and regional aquatic and terrestrial ecosystems that may otherwise be disconnected from the sources of the N gases. Food production systems are important contributors to local, regional, and global $NH_3$, $NO_x$ (NO + $NO_2$), and $N_2O$ budgets. Emissions of $NO_x$ contribute to local elevated ozone concentrations, while $N_2O$ emissions contribute to global greenhouse gas accumulation and to stratospheric ozone depletion.

Ammonia is the major gaseous base in the atmosphere and serves to neutralize about 30% of the hydrogen ions in the atmosphere. From terrestrial systems, 50 to 75% of the ~ 55 Tg $NH_3$-N year$^{-1}$ is emitted from animal and crop-based agriculture as animal excreta and synthetic fertilizer application. About half of the ~50 Tg $NO_x$-N year$^{-1}$ emitted from the earth's surface annually arises from fossil fuel combustion, and the remainder arises from biomass burning and emissions from soil. The $NO_x$ emitted, principally as NO, reacts rapidly in the atmosphere and in a complex cycle with light, ozone, and hydrocarbons, and produces nitric acid and particulate nitrate. These materials can interact with plants and the soil locally or be transported from the site and interact with atmospheric particulate to form aerosols. These salts and aerosols return to fertilize terrestrial and aquatic systems in wet and dry deposition. A small fraction of this N may be biologically converted to $N_2O$. About 5% of the total atmospheric greenhouse effect is attributed to $N_2O$ from which ~70% of the annual global anthropogenic emissions come from animal and crop production.

## FUTURE IMPACTS ON TERRESTRIAL AND AQUATIC SYSTEMS

### Atmospheric Deposition

The coupling of increased population with a move of a large sector of the world population to diets that require more energy and N input will lead to continued increases in anthropogenic input into the global N cycle. This scenario suggests that emissions of $NH_3$, $NO_x$, and $N_2O$ from agricultural systems will continue to increase and impact global terrestrial and aquatic systems, even those far removed from agricultural production, to an ever growing extent, unless N resources are used more efficiently or food consumption trends change. Increased N deposition on forests and other ecosystems with relatively closed N cycles has the potential to drive these ecosystems to more open states for their internal N cycles. This effect is illustrated where increases in N emission and deposition, which have occurred in industrialized regions of the northern hemisphere and which are projected to continue into the future across more areas of the world, could diminish the degree of N limitation of forest growth, alter forest community composition, change plant-soil interactions, and open forest N cycles. These responses to N deposition have implications not only for forests, but also for the atmosphere and for ecosystems that receive resulting nutrient outputs from forests. The gaseous redistribution of N has had and is having profound impacts on the quality of terrestrial and aquatic ecosystems and on the atmosphere. Deposition of N can stimulate productivity in N-limited grasslands, forests, and aquatic systems.

### On-Farm Needs

Available N from soil, fertilizer, and manure sources is inefficiently used (30 to 60%) in most crop production systems. As a consequence, unused inorganic N can move off crop fields and contaminate surface and groundwater resources. Governments have responded with guidelines, standards, regulations, and in some cases, fines when off-field losses of N have not been reduced. Numerous technologies and time-proven practices are available for producers to employ that will result in improved crop N-use efficiency (NUE). There are currently various emerging prediction and management technologies to help minimize and control impacts of N in the environment. Opportunity for improvement at the farm level largely lies with technologies that enable timely and accurate measurement of the spatial variability of crop yield potential, soil N availability, and within-season indication of crop N health. Soil-N excess and deficiency often exist on the same field. Thus, it is the variability in space and time of the processes that regulates the availability of N to plants and the fate of N in soil, making predictive technologies and precision N management attractive. On-farm technologies and practices to improve the NUE in agricultural production systems are becoming available to help producers make better N management decisions and to facilitate their capability to implement such changes.

Among the on-farm technologies and best management practices (BMPs) to improve NUE include the following: N recommendations based on determination of crop requirements, use of historical and realistic target yields, precision farming technologies (yield mapping and remote sensing), use of soil testing and assessing plant-available soil N, plant measurements (leaf/canopy greenness, spectral reflectance), N and other nutrient budgets, Internet and computer modeling tools, downloadable spreadsheets and applications, advanced computer software for livestock manure management, computer databases, livestock manure-management planning, and Internet natural resources databases (soil survey, etc.). Prerequisites for grower adoption require that technologies and practices be reliable, incur minimal additional expense (time and equipment), and integrate with ease into current operations. When these criteria cannot be met, external incentives (e.g., regulation, private or government cost-sharing programs, etc.) may be necessary.

### Computer Models and Nutrient-Management Planning

Computer models can serve as tools to assist with on-farm management decisions to aid in improving the NUE from livestock manures, for water quality prediction and protection, and to aid in the use of the BMPs of crop production. Prediction models can assist in identifying vulnerabilities of N and greenhouse gas emissions. Increasingly, crop and livestock producers will be required to develop nutrient-management plans to demonstrate that their operations have sufficient crop acreage, seasonal land availability, manure-storage capacity, and application equipment to manage animal manures, commercial fertilizers, and other land-applied nutrient resources in an environmentally responsible manner. Computer software has been and will continue to be used to help develop these plans. New nutrient management planning software needs to increasingly address the temporal and spatial nature of nutrient management, provide ways to accommodate regional areas and changing regulatory reporting requirements, utilize national databases and standards, and take advantage of modern software technologies, including those that are “Internet” based.

Potential scenarios for cropping systems, N, and/or water management will inherently be developed in cooperation with local producers, commodity, and action agency groups. However, comparisons among simulations of various management scenarios should take into account the uncertainty in the results obtained from calibration and validation studies. Larger potential differences should be targeted when selecting management scenarios to be tested. Much progress is occurring to develop computer modeling to describe gaseous $N_2O$ emissions. Currently, simulations are primarily being used to explore how $N_2O$ gas emissions respond to changes in land management, soil texture, and precipitation and to describe how annual $N_2O$ emissions can be reliably simulated for some native and managed systems. The mitigation of N gas losses from soils is strongly dependent on land management, but generalizations based solely on soil N and water inputs are limited because of the additional great importance of soil texture, soil organic matter levels, and timing of management practices. Low N gas emissions have been observed and simulated from soils under native vegetation, intermediate emissions from dryland agriculture, and high emissions from irrigated agricultural soils. Seasonal patterns of N gas emissions within systems and differences in mean emissions among systems have been simulated for native range grass, winter wheat/fallow conventional till and no till, winter wheat/corn/fallow no-till, and irrigated corn and silage cropping. Soil water inputs, tillage, timing of crop/fallow periods, and fertilizer application interact to control N gas emissions so that generalizations regarding land use are difficult to make.

NUE in production agriculture is often too low and the resulting losses of excess N to groundwater as $NO_3^-$, to gaseous emissions of $NH_3$ and $N_2O$, and to surface runoff and erosion are too high. Field studies designed to investigate potential BMPs are both time consuming and costly and cannot cover all scenarios. Application of simulation models with N cycling components in conjunction with associated field investigations offers methodology that can help identify on-farm BMPs that show promise in increasing NUE, but at reduced cost and time expended. Credible BMP studies employing simulation tools need to proceed along a well-defined path involving model selection, model adaptation and calibration, sensitivity analyses, data requirements and availability, model application, and model result interpretation and limitations. Early and continuing interaction with local producers and field research programs is an essential part of these BMP modeling studies.

Computer simulation and decision support (DSS) models for soil-crop systems that emphasize the N cycle, especially when coupled with economics and geographical information systems (GIS), are viable alternatives that can contribute to evaluating different combinations of management scenarios and how they impact the recovery of N by a cropping system for a given set of conditions. These models represent a complex series of algorithms and databases that can interact with different conditions and serve as mechanistic tools to evaluate different scenarios and their effects on NUE and the sustainability of a system. Some examples of more recent (since 1991) models that can be used to simulate C/N dynamics are the Root Zone Water Quality Model, RZWQM[7]; the Nitrate Leaching and Economic Analysis Package, NLEAP[8]; the Great Plains Framework for Agricultural Resource Management, GPFARM[9]; GLEAMS[10]; the CENTURY carbon model[11]; the Danish Nitrogen simulation system, DAISY[12]; the Rothamstead N turnover model, SUNDIAL[13]; the German UFZ model, CANDY[14]; the Canadian model, ecosys[15]; the Swedish model, SOILN[16]; and the Dutch model, ANIMO[17]. Many of these and other models have Web sites that contain model descriptions and, in some cases, the latest versions of the models and their associated databases.

### DRINKING WATER SUPPLIES

Nitrate contamination of drinking water supplies can result from a number of activities. More intensive crop production has caused an increase of $NO_3^-$ derived from animal wastes or fertilizers, which migrate slowly downward through the overlying soils to aquifers or by overland and interflow processes to surface waters used as drinking water supplies. Because of its importance and widespread occurrence, remediation of high-$NO_3^-$ drinking water supplies for rural and urban populations is potentially an increasingly important issue.

#### Rural Water Supplies

In rural areas, agricultural activities that involve the use of fertilizers and animal manures are major sources of $NO_3^-$ contamination. Nitrate is the most common N contaminant in raw water supplies. Several processes are currently available that can effectively remove $NO_3^-$ from raw water. Systems that are suitable for small rural communities include reverse osmosis, ion exchange, and electrodialysis. However, all of these systems remove $NO_3^-$ from raw water and concentrate it in a reject water or brine. Disposal of the reject water can be a major expense as well as an environmental issue. Several emerging systems that convert $NO_3^-$ to harmless $N_2$ gas are under development. These include biological denitrification systems and catalytic systems. The ability of these systems to convert the $NO_3^-$ to $N_2$ gas is a major advantage. Although more or less applicable to specific remediation needs, reverse osmosis, ion exchange, and distillation can be used in home systems to remove $NO_3^-$ from raw water. Reverse osmosis and distillation are small point-of-use systems that provide water for cooking and drinking. In the future, biobarriers might be used to protect a well from a contaminated aquifer or to protect an aquifer used for drinking water from a source of $NO_3^-$ pollution. Aboveground denitrification reactors might be used to provide $NO_3^-$-free water to rural communities in the not-too-distant future.

#### Urban Water Supplies

For most cities where high-$NO_3^-$ drinking water supplies exist, establishing alternate supplies of water is most cost-effective. Generally, $NO_3^-$ is a problem in surface or shallow groundwater supplies. Drilling a new, deeper well may also be an alternative for some cities. However, another alternative is the one being used by the City of Des Moines Water Works in Iowa (U.S.) to serve approximately 350,000 people and where $NO_3^-$ concentrations from the city's water supplies had risen to a point where other alternatives were not feasible. Ion exchange using a strong anion resin was the most desirable treatment process.

### PUBLIC POLICY CONSIDERATIONS

Development of policy to control unwanted reactive N release is difficult because much of the release is related to food and energy production[6], and reactive N species can be transported great distances in the atmosphere and in aquatic systems. Reducing new reactive N and curtailing the movement for food and energy production systems is especially difficult because many countries need to keep up with population growth, or at least raise nutritional levels. Multinational efforts to control N loss to the environment are very much needed. However, the following considerations may help to illustrate policies associated with some of the issues that relate to this important topic.

#### Social and Biophysical Processes

In the future, numerous economic and policy issues will need to be addressed. The users and producers of reactive N live not only in biophysical space but also in social space. Salient social and biophysical processes are not random in space or time. As an example, social factors that influence farmers arise at multiple and overlapping scales in an agro-environmental hierarchy. Conceptually, a parallel hierarchical statement can be made concerning the energy and transportation sectors of society. Inappropriate behaviors at vulnerable locations or times create many of the "problems" to which policy and research agendas are oriented today. This situation of "disproportionality" sends a warning that there is not a universal policy or technical solution to the problem of N leakage into the environment, and it is necessary to first be able to describe and measure pattern and process before seeking explanation and prediction. Concepts such as scale and context apply. Management and therefore mismanagement is a cross-scale phenomena, the resultant social process of many different actors and institutions, including but not limited to the primary users.

In the case of agriculture, the issue of studying linkages between the social and biophysical factors affecting N management points to the need for a common area of analysis. There is significant variation in nutrient management behavior of farmers that leads to a disproportionate influence on ecosystem properties. Examining the role of disproportionality will involve linking social phenomena to biophysical phenomena at different spatial and temporal scales. Spatial-analysis methodology using common analytical techniques and research on N processes as well as both social and biophysical data to describe disproportionality at various spatial scales is required.

#### Policy Development

Continuing the discussion and with agriculture as an example, advances in linking the science of ecology with economics and the development of agro-environmental modeling systems will require that they be coupled with new information technology. New public policy approaches may well need to reward agricultural producers for providing ecological services to advance the social and economic agendas of the public and of policymakers. Such a model to simulate complex relationships among agricultural land management practices, economic costs, and environmental impacts allows for commoditization of services, and hence the possibility of new markets. Markets, however, require clarity regarding property rights to what is traded. Government needs to define what it is that is traded with the use of these models, help establish who owns the product of the services (as well as who is liable for nonperformance of service and the extent of liability in many cases), and establish conditions for monitoring and enforcement where the primary beneficiary of the service is the public.

Finally, to those who are concerned with the environmental, policy, or economic issues, the need exists for all to recognize that agriculture as a business is conducted outdoors and faces considerable risk and adversity as a result of the vagaries of climate each year. Depending upon location and year, climatic vagaries can include regional heat waves, droughts, severe storms, and/or flooding. From an economic perspective, the purpose for the use of N in agriculture is to increase crop yields, improve the economic return realized by the producer, and foremost to meet human needs for food and dietary protein. To address the issue of N in the environment, which is in fact increasingly becoming a complex societal (and global) problem, will require a sustained dialogue among policymakers, scientists, practitioners, and affected stakeholders. The interaction of scientists and policymakers, in particular, often does not occur in a regular or orderly manner to help benefit policymaking. In general, the more complex the problem is, which may require legislative solutions, the longer and more deliberative the process to craft the legislation must be. In the case of issues with a complex scientific underpinning, such as the issues associated with N in the environment, the deliberative process will undoubtedly require a thorough, thoughtful interface between scientists, who tend to speak in terms of "uncertainties," and policymakers, who seek bottom-line "certainties" necessary to create good policy and prudent laws.

### ACKNOWLEDGMENTS

The authors especially and gratefully acknowledge the efforts of the chapter authors who contributed to the book edited by Follett and Hatfield (2001) and the information available from the chapters developed by the individual authors of those chapters.

| | | |
|---|---|---|
| Received: | July | 4, 2001 |
| Revised: | September | 25, 2001 |
| Accepted: | October | 3, 2001 |
| Published: | October | 30, 2001 |
